# Knowledge, attitudes, and practices on the use of local anaesthesia in the Emergency Department

**DOI:** 10.4102/jcmsa.v2i1.85

**Published:** 2024-10-16

**Authors:** Diana B. Dickinson, Craig Beringer, Pravani Moodley

**Affiliations:** 1 Division of Emergency Medicine, Faculty of Health Sciences, University of the Witwatersrand, Johannesburg, South Africa

**Keywords:** local anaesthetic, local anaesthetic systemic toxicity, LAST, Intravenous lipid emulsion therapy, ILE

## Abstract

**Background:**

Local anaesthesia (LA) is frequently used in the Emergency Department (ED). Local anaesthetic systemic toxicity (LAST) is a potentially fatal complication associated with its use. It is assumed that all doctors administering LA are trained in all aspects of its use including the management of LAST.

**Methods:**

A prospective, multicentre cross-sectional study was conducted at three academic EDs in Gauteng. Doctors working in the ED completed an online questionnaire designed to assess knowledge, attitudes and practices regarding the use of LA as well as the recognition and management of LAST.

**Results:**

A total of 94 completed questionnaires were analysed. Of these, 44% of participants had graduated from medical school less than 5 years ago, and 72% had less than 5 years of experience working in the ED. The overall mean knowledge score percentage for the safe use of LA was 48.2%. The overall mean knowledge score percentage for recognition and management of LAST was 56.7%. A total of 78% of participants knew that intravenous intralipid emulsion (ILE) therapy is needed in managing LAST; however, only 25% of participants knew where to access ILE and only 33% knew how to use it.

**Conclusion:**

The overall level of knowledge on LA and the management of LAST among doctors working in the ED is low. There is also an apparent lack of awareness of the potential for toxicity despite frequent use of LA. Although the occurrence of LAST is rare, our findings highlight an obligation for continuous education on local anaesthesia and the management of LAST.

**Contribution:**

The result of this research indicates a gap in training regarding the potential for toxicity of local anaesthesia, recognition of systemic toxicity, and the management thereof. These findings suggest the possibility of misdiagnosis and under reporting of LAST.

## Introduction

South Africa (SA) has one of the highest trauma-related morbidity and mortality rates in the world.^1,2^ This trauma burden is responsible for up to 40% of South African Emergency Department (ED) attendances each year.^2^

Local anaesthesia (LA) is often used when managing trauma patients, with usage ranging from local infiltration for minor wound care to ultrasound guided nerve blocks for the management of more extensive injuries.^3,4,5^

Commonly used LA agents in the ED include lignocaine and bupivacaine, with bupivacaine having a higher risk of toxicity.^6^ The correct use of LA, which includes routine accurate weight-based dosing, is imperative to ensure the avoidance of complications, most notably that of local anaesthetic systemic toxicity (LAST).^4^

Local anaesthetic systemic toxicity, a potentially fatal complication related to the use of LA, was first recognised almost 44 years ago.^7^ The recognition and management of LAST requires awareness and basic monitoring. Symptoms of LAST affect the central nervous system (CNS) and the cardiovascular system (CVS), with certain populations (extremes of age, pregnancy, those with cardiac, renal and/or liver disease) being at a higher risk of developing toxicity than others.^8,9,10^ Central nervous system manifestations range from early, prodromal excitatory symptoms to more severe symptoms including altered mental state, seizures and respiratory depression.^8,11^ Cardiovascular system symptoms are usually associated with more severe toxicity and may result in dysrhythmia’s, haemodynamic instability and cardiac arrest.^8,11^

In 2010, the American Society of Regional Anaesthesia and Pain Medicine developed one of the first practice advisories on the safe use of local anaesthesia and the management of LAST, recommending intravenous intralipid emulsion (ILE) as the cornerstone in management.^9^

Despite advances in knowledge regarding the development and management of LAST, case reports published between 2010 to date indicate that LAST continues to occur at an estimated rate of 1.8/1000 nerve blocks.^10,12,13,14,15,16,17^ In addition, the literature suggests that due to the likelihood of under-reporting or misdiagnosis, the true incidence of LAST may be underestimated.^10^

It is expected that all doctors using LA have sound knowledge on dosing as well as on the recognition and management of complications, most specifically LAST. A review of the literature, however, has highlighted a consistent gap in knowledge of both the safe use of LA and the management of LAST with specific relation to level of experience.^3,18,19,20,21^

To our knowledge, there have been no studies to assess doctors’ knowledge, attitudes and practices of LA use and the management of LAST in SA. The aim of this study was therefore to assess this in a South African setting.

## Research methods and design

### Study design and Setting

A prospective, multicentre cross-sectional study was conducted.

Data collection occurred over a 2-month period (01 March 2023 – 30 April 2023) in three academic state hospitals in Gauteng, affiliated to the University of the Witwatersrand.These included one tertiary-level facility: Helen Joseph Hospital (HJH) and two regional-level facilities: Tambo Memorial Hospital (TMH) and Thelle Mogoerane Regional Hospital (TMRH).

### Study population

The study population was based on convenience sampling and consisted of 120 doctors working in the specified ED’s at the time of data collection. This included interns, community service medical officers, medical officers and emergency medicine registrars. Specialist physicians and doctors who reported that they did not use LA in their practice were excluded.

### Data collection

Data was collected using a questionnaire adapted and developed from previous studies (Online Appendix 1).^3,18,19,20,21^ The data collection instrument used was designed to assess three subscales including knowledge, attitudes and practices.^22^

The questionnaire included sections on demographics (level of experience); LA use and availability; LA dose calculations; recognition (signs and symptoms) and management of LAST; as well as training received on the use of LA and management of LAST.

Knowledge-based questions were scored as ‘1’ or ‘0’ for each correct or incorrect answer, respectively. For the questions relating to attitudes and practices, these were assessed using Likert scales. Google Forms was used as the design tool for administering the questionnaire.

Participants accessed the questionnaire on their smart devices via a link or a quick response (QR) code provided to them. The questionnaires did not include any identifying information, with participants remaining anonymous throughout data collection. Questionnaires were completed and submitted online, automatically populating an Excel spreadsheet.

Data collection was performed via a face-to-face method to reduce the likelihood of participants discussing the questions or exiting the link to research answers. This ensured the most accurate reflection of the participants’ current level of knowledge. Calculators were provided where needed.

### Data analysis

Completed questionnaires were captured into an Excel spreadsheet. Data was exported and analysed using IBM^®^ SPSS^®^ Statistics, version 28.

The doctors’ levels of experience and LA usage patterns were shown in frequencies and percentages. Knowledge on the use of LA and management of LAST was computed, with the highest possible score of 100%, and is reported in means with standard deviation (s.d.).

One way analysis of variance (ANOVA) was used to examine if LA knowledge scores varied significantly according to the doctors’ levels of experience (i.e. < 5 years vs. > 5 years).

The Pearson’s point-biserial correlation coefficient was used to measure the strength and direction of the association between the doctors’ knowledge on the use of LA and management of LAST, with their level of experience. Other bivariate analyses involving categorical variables used the Pearson chi-square test of independence or, where the contingency tables had cells with less than five counts, the Fisher’s exact test instead.

All statistical analyses were considered significant for *p*-values < 0.05.

### Ethical considerations

Ethical clearance to conduct this study was obtained from the University of the Witwatersrand Human Research Ethics Committee (No. M221020 MED22-08-053). Written informed consent was obtained from all participants. Consent forms did not include any identifying information and participants remained anonymous.

## Results

Of the sample population of 120 potential participants, 98 consent forms were signed, and a total of 94 questionnaires were completed. The response rate was 96%. All completed questionnaires were included; none were excluded because of being incompletely filled.

### Demographics: Participants’ years of experience

The majority of participants had graduated from medical school more than 5 years ago, however, most of the participants had less than 5 years of experience working in the ED (Table 1).

**TABLE 1:** Participants’ descriptive information.

Parameter	*n*	%
Total number of participants	94	-
**Number of years since graduation**		
< 5 years	40	43
> 5 years	54	57
**Number of years in ED**		
< 5 years	68	72
> 5 years	26	28

ED, Emergency department.

### Frequency of local anaesthesia use

**FIGURE 1:**
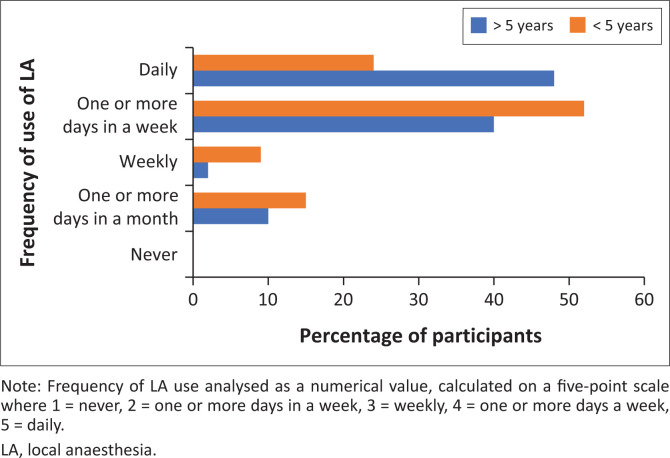
The frequency of local anaesthesia use with regard to years since graduation.

The mean use of LA among those with < 5 years since graduating medical school was 4.3 (s.d. = 0.9) compared to 3.9 (s.d. = 1.0) for those with > 5 years (*p* = 0.047) (Figure 1).

The mean use of LA among those with < 5 years working in the ED was 4.1 (s.d. = 1.0) compared to 3.9 (s.d. = 0.9) for those with > 5 years (*p* = 0.40).

The most frequently used LA agent was lignocaine (100%).

### Current level of knowledge on safe use of local anaesthesia

**FIGURE 2:**
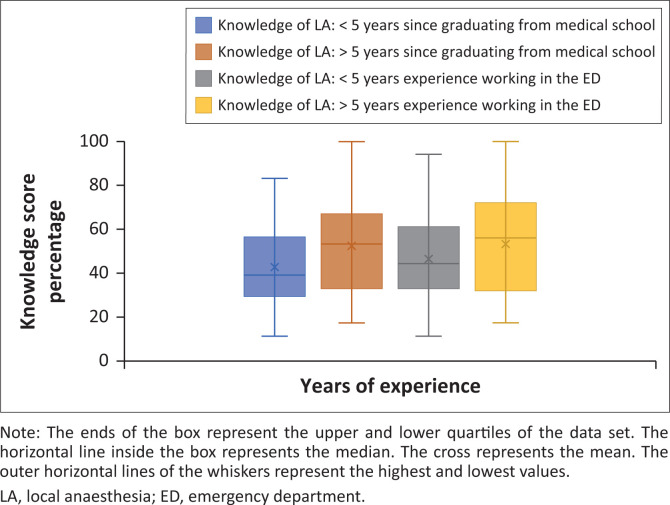
A box and whisker plot showing the knowledge score percentage on the safe use of local anaesthesia compared to years of experience (years since graduating medical school and years of experience working in the emergency department).

The overall mean knowledge score percentage for the safe use of LA was 48.2% (95% confidence interval [CI]: 44.3–52.1). A weak association was demonstrated between the mean knowledge score percentage and the number of years since graduating medical school (*r* = 0.24; *p* = 0.02). This association was also demonstrated with the number of years working in the ED (*r* = 0.15; *p* = 0.14) (Figure 2).

When assessing the knowledge of the maximum safe dose of lignocaine, less than half of participants (42%) gave the expected response (4.5 mg/kg) and 11% selected an unsafe, potentially toxic dose. The majority of participants (87%), however, provided answers that were within a safe dose range (1 mg/kg – 4.5 mg/kg).

Regarding bupivacaine, less than a third of participants (31%) knew the maximum safe dose of plain bupivacaine, and 29% knew the maximum safe dose of bupivacaine with adrenaline. Only 53% of participants knew that bupivacaine is more cardiotoxic than lignocaine.

### Current level of knowledge on recognition and management of local anaesthetic systemic toxicity

**FIGURE 3:**
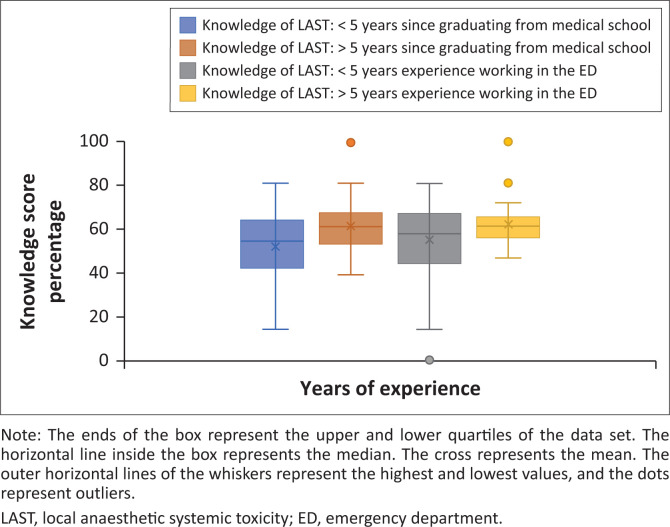
A box and whisker plot showing the knowledge score percentage on recognition and management of LAST compared to years of experience (years since graduating medical school and years of experience working in the emergency department).

The overall mean knowledge score percentage for recognition and management of LAST was 56.7% (95% CI: 53.6–59.8). A weak association was demonstrated between the mean knowledge score percentage and the number of years since graduating medical school (*r* = 0.35; *p* < 0.001). This association was also demonstrated with the number of years working in the ED (*r* = 0.23; *p* < 0.03) (Figure 3).

Regarding the risk factors that predispose patients to LAST, less than two-thirds of participants (60%) were aware that dose adjustment is necessary in patients with cardiac, liver or renal disease. Moreover, only 29% and 42% were aware that dose adjustment is necessary in pregnancy and in extremes of age, respectively.

Concerning the management principles of LAST, only 12% of participants knew that cardiac arrest in LAST is not solely managed according to standard Advanced Cardiac Life Support (ACLS) protocols. Worryingly, 52% of participants incorrectly reported that lignocaine can be used for refractory VF.

Comparatively, a large number of participants (78%) knew that intravenous ILE is needed in the management of LAST; however, only 33% of participants had read guidelines on how to administer it. A third of participants (33%) reported that they knew how to administer ILE but have never had to, with only 25% being aware that the ED in which they worked had access to it.

Thirty participants (32%) knew whether the ED in which they worked had a protocol on LA use or LAST management, and only 19% of participants were aware of any international guidelines on the safe use of LA and LAST.

### Current attitudes regarding the use of local anaesthesia and the management of local anaesthetic systemic toxicity

All participants agreed that doctors working in ED’s should be familiar with the pharmacology and side effects of LA agents. A total of 91 participants (97%) agreed that all doctors in the ED should be comfortable with managing adverse events relating to LA agents, including LAST.

Regarding undergraduate training on the use of LA and LAST, 72% of participants reported they had received training on the safe use of LA; however, only 38% stated they had received training on LAST.

### Current practices regarding the use of local anaesthesia and the management of local anaesthetic systemic toxicity

**FIGURE 4:**
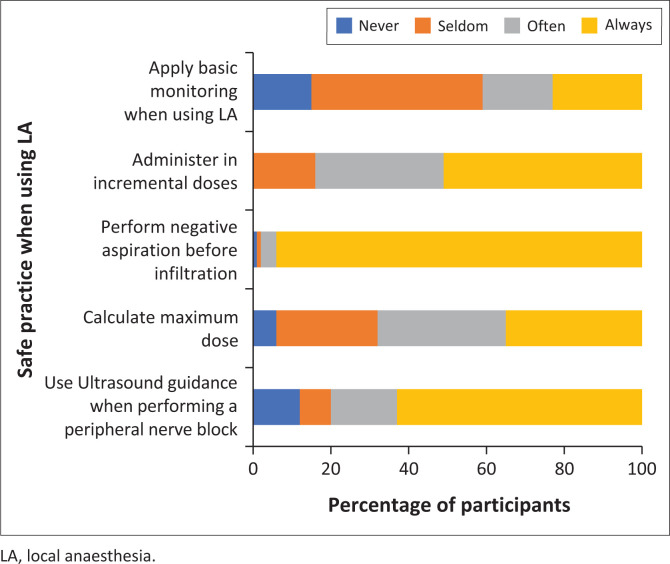
The practices of doctors in the emergency department when using local anaesthesia.

A significant amount of participants (32%) seldom or never calculate the maximum dose of LA before administration (Figure 4).

Twenty-two participants (23%) reported that they always applied basic monitoring when administering LA. A negative correlation was found between the frequency of LA use and the use of monitoring (*r* = –0.23; *p* = 0.01).

With regard to the use of ultrasound (U/S), 63% of participants reported that they always use U/S guidance when performing a peripheral nerve block (PNB). The remainder reported use of U/S guidance as, often (17%) seldom (8%) and never (12%). A positive correlation was found between the use of U/S for PNB and the number of years working in the ED (*r* = 0.20; *p* = 0.048).

Pertaining to the use of ILE, only 42% reported that they would feel comfortable administering it. Six (6%) participants reported having witnessed or been involved in the management of a case of LAST and only three participants reported having used ILE.

## Discussion

Local anaesthesia use in the ED is not limited to wound care, but forms part of the armamentarium of multimodal analgesia. Although this was a small study looking at doctors working in the ED, the findings emphasise a gap in knowledge regarding the safe use of LA and the management of LAST.

Although bupivacaine remains the more toxic LA agent, recent literature has highlighted an increase in cases of LAST related to the use of lignocaine.^23^ In our study, the most frequently used LA agent in the ED was lignocaine.

The maximum reported safe dose of LA agents remains inconsistent. Munasinghe et al. demonstrated a significant difference in the knowledge on the maximum dosing of LA among doctors in Sri Lanka. They found that only 58% of participants correctly identified the maximum safe dose of plain lignocaine.^18^ Our findings were similar in that only 42% gave the expected response (4.5 mg/kg); however, a total of 87% would have administered a dose within the safe range. The lack of consensus on the maximum safe dose quoted in literature, which ranges between 3 mg/kg and 5 mg/kg could account for this.^8,11,24,25,26^

In 2010, Cooper et al. conducted the first study in an ED looking at knowledge of doctors on LA and LAST. They found a concerning gap in the knowledge of junior doctors regarding safe dosing of LA and the subsequent management of toxicity.^3^ Comparably, our study demonstrated a similar gap in knowledge among more junior doctors, who according to our findings also used LA more frequently. This highlights a gap in undergraduate training, with 28% and 62% of participants reportedly having not received training on the safe use of LA and the management of LAST, respectively.

The use of U/S guided PNB has been shown to decrease the risk of developing LAST.^4,26^ The ED’s in our study all have a functional ultrasound machine available for use in the unit. Despite this, only 63% reported that they always use U/S guidance when performing a PNB with a positive correlation found between the level of participant experience and the use of U/S. This may be because of low level of confidence with the use of U/S by less experienced doctors and suggests a need to improve training of doctors working in the ED regarding U/S guided procedures.

A Turkish study assessing the knowledge of LAST among emergency physicians (EPs) found that the knowledge among EPs faired higher when compared to other studies; however, the overall awareness of LAST remained low. They reported that only 20.8% of EPs identified all symptoms of LAST and 22.5% answered all questions related to treatment correctly.^19^ In spite of the fact that their study looked at EPs and ours had a more heterogenous population that excluded specialist physicians, we found that although participants with more experience had higher scores, the overall knowledge in both groups was low. These findings imply a need for continuous education on LA and the management of LAST.

Detection of LAST requires basic monitoring including non-invasive blood pressure, electrocardiography, and pulse oximetry measurements. Only 23% of participants in our study reported that they always used monitors when using LA, with a negative correlation indicating that those who used LA more frequently were less likely to apply monitoring. A possible explanation for this low utilisation of monitors could be resource limitation. This finding suggests the potential for under-reporting and misdiagnosis of LAST.

In 2018, the American Society of Regional Anaesthesia and Pain Medicine published updated guidelines on the management of LAST.^23^ Despite this, only 19% of participants were aware of any international guidelines on the management of LAST.

The development and use of protocols in the ED is known to improve the diagnosis and management of emergencies.^27,28^ A lack of ED protocols on the safe use of LA and management of LAST, coupled with a poor awareness of LAST, can contribute to an increased risk for LAST.

Given the high frequency of use of LA, it is imperative that all doctors working in an ED in SA are proficient in the safe administration of LA and are well versed in both the prevention and management of LAST. The results of our study demonstrate an overall deficit in knowledge and a need for guidance with regard to safe use of LA and LAST management.

### Limitations

This was a knowledge-based survey, which is subject to recall bias. Convenience sampling may have resulted in both sampling and selection bias. Face-to-face recruitment may have increased the risk of response bias as participants with greater knowledge may have been more likely to participate. Although it was a multicentre study, the study population was quite small. Only doctors working in academic hospital emergency departments were included, and thus our results may not be generalisable.

## Conclusion

The overall level of knowledge on LA and the management of LAST is low among doctors working in the ED, with a lack of awareness towards the potential for toxicity despite the frequency of use. This suggests the possibility of misdiagnosis and under-reporting of LAST. Although the occurrence of LAST is rare, our findings highlight an obligation for continuous education on the use of LA and the management of LAST. In addition, there is an apparent need for the development of local guidelines. We propose that all EDs should develop protocols on the safe use of LA, the recognition of LAST and the management thereof.

### Dissemination of results

The results of this study will be shared with the head of the ED at each of the data collection sites and participants. This will be done through an informal presentation with the intention of promoting continuous education on the topic.
